# A tough and mechanically stable adhesive hydrogel for non-invasive wound repair

**DOI:** 10.3389/fbioe.2023.1173247

**Published:** 2023-04-13

**Authors:** Xiaochun Liu, Si Qin, Lei Xu, Guo Fu, Yongjun Huang, Chaoqun Yu, Guoyun Cheng, Ying Li, Yunzhi He, Yong Qi, Dawei Sun

**Affiliations:** ^1^ Department of Orthopedics, Guangdong Second Provincial General Hospital, Guangzhou, China; ^2^ Department of Dermatology, Guangdong Second Provincial General Hospital, Guangzhou, China; ^3^ Department of Orthopedics, Sun Yat-sen Memorial Hospital of Sun Yat-sen University, Guangzhou, China

**Keywords:** adhesive hydrogel, topological adhesion, wound repair, toughness, mechanical stability

## Abstract

Wound healing has been a great challenge throughout human history. Improper treatment for wounds is so easy to lead to infection and a series of serious symptoms, even death. Because of the ability of absorbing fluid and keeping a moist environment, the hydrogel with 3D networks is ideal candidate for wound dressing. More important, it has good biocompatibility. However, most of the hydrogel dressings reported have weak mechanical properties and adhesion properties, which greatly limit their clinical application. Herein, a tough adhesive hydrogel with good mechanical stability for non-invasive wound repair is reported. The hydrogel is composed of polyethylene glycol dimethacrylate (PEGDA), chitosan (CS) and chitin nano-whisker (CW). PEGDA and CS form interpenetrating network hydrogel through free radical polymerization reaction under the UV light. The introduction of CW further enhances the toughness of the hydrogel. The pH-sensitive CS can form adhesion to various materials through topological adhesion. As a wound closure repair material, PEGDA/CS/CW hydrogel not only has the characteristic of effectively closing the wound, defending against invading bacteria, and keeping the wound clean, but also has good tensile and mechanical stability, which is expected to realize the closure and repair of joints and other moving parts of the wound. This adhesive hydrogel is proven a promising material for wound closure repair.

## 1 Introduction

Skin is the most important organ covering the surface of the human body and contacts with the external environment in direct. And it is an important barrier to protecting the body from the external environment. It also has functions such as feeling external stimuli, regulating body temperature, preventing water loss in the body and protecting the body from external injuries (Montagna, 2012; Vig et al., 2017; Dąbrowska et al., 2018; Liang et al., 2021). But every year, even every day, tens of thousands of people suffer intentional (surgery) or unintentional (burns or cuts) damage to skin tissue. Wound tissue healing is a dynamic and complex process, which is one of the human body’s most complex biological processes. Improper treatment of wounds leads to increasing healing time and a higher risk of infection (Gonzalez et al., 2016; Du et al., 2019; Du et al., 2020; Liu et al., 2023). There are various types of wound dressings constantly being researched and developed that can make wounds heal faster and more effectively. As we known, the traditional wound dressings (gauze and cotton) are often used in clinical treatment, but these dressings are so poorly self-adhesive that need to be fixed with external objects. Moreover, it is easy to accumulate exudate or blood and stick to the wound tissue, that causes secondary bleeding and pain during dressing change (Dhivya et al., 2015; Li et al., 2019). In recent years, several new dressings such as film (Chang et al., 2022; Pitpisutkul and Prachayawarakorn, 2022), foam (Patil et al., 2022), hydrogel (Liang et al., 2021; Zeng et al., 2022) and hydrocolloid dressings (Phillips et al., 1994; Kong et al., 2020) have attracted increasing attention, because these new dressings can provide a moist, antibacterial healing environment and actively promote the wound healing process (Zhang et al., 2020). Among them, hydrogel dressing shows outstanding advantages in the field of wound dressing due to its excellent biochemical and mechanical properties (Liang et al., 2021).

Hydrogel with adhesive properties have a great biomedical application prospect. So far, there is no shortage of experts and scholars to develop a series of adhesive hydrogel for wound repair, to make up for the shortcomings of needle and thread suture and traditional dressing (Hu et al., 2019; Yang et al., 2021). Cyanoacrylate is a strong adhesive material but cytotoxic after adhering and hardening the interface of materials (Vakalopoulos et al., 2015). Fibrin and polyethylene glycol gels are flexible, but them have weak adhesion strength (Annabi et al., 2015). Hugo Schiff proposed the reaction between the aldehyde or ketone group and amino group to produce an imine bond, which has been widely used in the preparation of adhesive hydrogel (Xu et al., 2019). Aldehyde hydrogel has mild reaction conditions and it forms rapid adhesion to tissues. However, it usually exhibits weak interface toughness, leading to limited applications (García and Smulders, 2016). The ideal wound dressing should be safe, tough, simple to prepare. Moreover, it should have good adhesion to fully fit the wound. Chitosan is a macromolecule with fine biosafety widely used in bioengineering (Kim et al., 2008; Sahariah and Másson, 2017; Minh et al., 2019). Chitosan has properties that accelerate wound healing, making it a prime candidate for possible control of skin-associated infections (Costa et al., 2018). In addition, the amine group on chitosan has a special reaction with the change of environmental pH value (Okuyama et al., 1997; Ladet et al., 2008). Thus, Suo et al. introduced a molecular suture material method (topological adhesion) due to chitosan’s pH-sensitive performance (Yang et al., 2018). Topological adhesion opens up a new area of development, enhancing the strength of adhesion between materials while maintaining the softness of materials.

**FIGURE 1:**
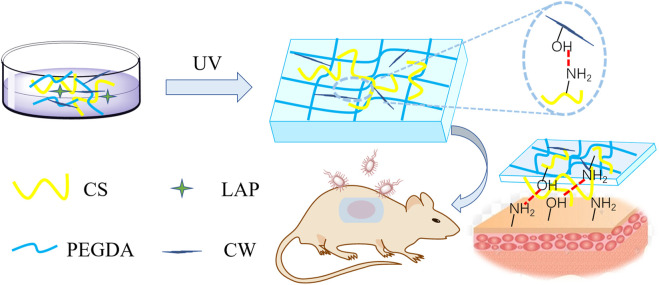
Structure scheme of the adhesive and elastic hydrogel for wound closure.

In order to make up for the traditional wound closure methods such as suture and wound closure device easy to bring secondary injury to the wound, a tough and mechanically stable adhesive hydrogel for non-invasive wound repair was constructed in this study, which could be applied to close and repair skin wounds (Figure 1). Polyethylene glycol dimethacrylate (PEGDA) hydrogel has outstanding mechanical stability, and the topological adhesion enables hydrogels to form strong adhesion to tissues without any chemical reaction with tissues. Chitosan (CS) and chitin whiskers (CW) enhance the toughness of hydrogel through molecular entanglement and hydrogen bonding. In addition, CS can freely diffuse and permeate through tissue interfaces and tightly adhere to surfaces of various materials including tissues. Topological entanglement and ion chelation contribute to improve the adhesive property. The hydrogel has excellent flexibility and toughness, can prevent bacterial infection, absorb tissue effusion and promote wound healing. Therefore, it has particular potential as a wound dressing in wound healing.

## 2 Experimental methods

### 2.1 Materials

The CS (*M*_w_ = 100,000 Da) was purchased from Macklin Biochemical Co. Ltd. (Shanghai, China). According to our previously reported protocols (Hu et al., 2018; Huang et al., 2019), the polyethylene glycol and acryloyl chloride were combined in anhydrous dichloromethane solvent under nitrogen atmosphere to create the PEGDA (*M*_n_ = 6,000 Da). Chitin (Ch, the degree of acetylation is 90%) was obtained from Shanghai Maclin Biochemical Technology Co. Ltd. Lithiumphenyl-2, 4, 6-trimethyl-benzoylphosphinate (LAP, the purity is 97%) was provided by Shanghai Bide Medical Technology Co. Ltd. The rest of the chemical reagents were all of analytical quality and were employed right away.

### 2.2 Preparation of chitin nano-whisker (CW)

The preparation steps of CW are as follows: 20 g of chitin powder were weighed in an electronic balance and placed in a dry three-way flask. Then, the concentrated hydrochloric acid (HCl) was diluted to 3 mol/L with deionized water. 75 mL 3 mol/L HCl was added into the bottle containing chitin powder. The mixed solution was stirred at 95°C for 150 min under N_2_ atmosphere. The heating was stopped at the end of the reaction. When the suspension in the three-way flask cooled naturally to room temperature, it was put into a 50 mL centrifuge tube and centrifuged at a speed of 5,000 r/min. Left a precipitate from the solution to remove the supernatant. The precipitation was suspended in deionized water, dispersed by ultrasonic, and centrifuged. The washing process was repeated many times until the supernatant was neutral after centrifugation. The precipitation after centrifugal washing was suspended in deionized water and then frozen in a −80°C refrigerator for 24 h. After 24 h, the precipitation was transferred to a freeze dryer for 24 h. Finally, the powder was grounded in a mortar to obtain CW powder, which was packed into a sample bag and stored in a drying cabinet.

### 2.3 Preparation of hydrogel

The hydrogel in this study was produced by free radical polymerization initiated by UV light. Firstly, a certain mass of CS was weighed with an analytical balance, and then dissolved in an acetic acid solution with a volume ratio of 1%. Finally, the CS solution with a mass volume ratio of 2% was prepared. Taken 10 mL of the prepared CS solution above into the centrifuge tube. Then 1.5 g PEGDA powder, 0.5 g CW powder and 0.025 g LAP powder were added, respectively. Followed by ultrasound for 20 min. When PEGDA and LAP powder were fully dissolved and the CW was evenly dispersed, the prepolymer solution was obtained. Finally, the prepolymer solution was poured into the silicone mold. The PEGDA/CS/CW hydrogel was obtained after being exposed to the ultraviolet crosslinker (365 nm, 50 mW/cm^2^) for 3 min. The synthesis methods of PEGDA hydrogel and PEGDA/CS hydrogel followed the similar procedure.

### 2.4 Fourier transform infrared spectrometer (FTIR) analysis

FTIR spectra was measured on the FTIR spectrometer (Nirolet, America, 6,700) at 25°C with the wave number of 500–4,000 cm^−1^.

### 2.5 X-ray diffraction (XRD) analysis and morphology observation

XRD (Shimadzu Corporation, Japan, XRD-6100) was used to examine the crystal structures of the Ch and CW by the Cu Ka target, voltage 40 kV, current 30 mA, scan range 2θ = 5°–60°, and scan rate 10°/min.

Transmission electron microscope (TEM, Rigaku Corporation, Japan, Philips TECNAI 10) was used to examine the morphology of CW by the voltage 40 kV. CW powder was dispersed in 50% ethanol, which the concentration was 0.01 wt%. After ultrasonic treatment for 20 min, a small drop of sample solution was suctioned by a pipette or dropper into a 200-mesh copper net, which was placed on filter paper for natural air drying. After drying completely, TEM was used to observe its morphology. The CW length in TEM images was measured by ImageJ software.

### 2.6 Swelling characteristics

The swelling properties of the hydrogel crosslinked by ultraviolet light were measured at 37°C, which was submerged in phosphate buffered saline (PBS, 0.1 M) with a pH of 7.4. Before performing the swelling test, the initial weight (*W*_i_) of hydrogel was noted. In the process of testing, the solution on the surface was periodically removed using filter paper and weighed (*W*_t_). The swelling ratio (*SR*) of the samples were calculated by Eq. 1 (Huang et al., 2019).

$$SR=\frac{W_{t}-W_{i}}{W_{i}}\times 100\%$$ (1)

### 2.7 Adhesion tests

Lap shear tests were used to assess the hydrogel’ adhesive properties. The rectangular porcine skins (60 × 30 mm) were sandwiched between the cuboid hydrogel samples (20 × 15 × 1.5 mm). The stretch separation test was performed after 2 min of gentle pressure with the finger. At a constant tensile speed of 2 mm/min, all experiments were conducted. The greatest load divided by the bond area was used to compute adhesion strength [13, 17, and 28].

### 2.8 Mechanical tests

A universal testing device was used for the mechanical tests (Shimadzu Corporation, Japan, AG-I, 100 N sensor).

The mechanical characteristics of the hydrogel were assessed using compression and tensile tests. The hydrogel samples were made into cylinders, which are 10 mm in diameter and 6 mm tall for the compression tests. It was 2 mm/min for a single compression. With 20 cycles and a compression strain range of 0%–50%, the cyclic compression rate was set at 3 mm/min. Cut the hydrogel samples into dumbbell shapes before performing the tensile testing (length 50 mm, width 4 mm, thickness 2 mm). The single stretch rate was set to 10 mm/min. The tensile strength of hydrogel is the maximum fracture stress in the stretching process of hydrogel (Liu et al., 2023). With 20 cycles and a tensile strain range of 0%–100%, the cyclic tensile rate was set to 50 mm/min. The compressive elastic modulus was obtained by fitting the 10%–40% strain region of the compressive stress-strain curve.

### 2.9 Cytocompatibility

Mouse fibroblasts (L929) were tested for biocompatibility. First, a cylindrical hydrogel with a diameter of 10 mm and a thickness of 2 mm was immersed in 75% ethanol for 30 min and UV irradiation for 1 h to sterilize the sample. Finally, the sample was washed three times with phosphate buffer (PBS). The sterilized samples were soaked in cell culture solution for 24 h to obtain an extract (concentration of 20 mg/mL). The cells were then cultured with extract (hydrogel group) and normal medium (control group), and the cell concentration was 1 × 10^4^ cells/mL. The cells were kept constant at 37°C, 5% CO_2_ and 95% relative humidity incubate in incubators. Cell viability was determined by the cell counting kit-8 reagent (CCK-8, biosharp, China) at 1, 3, and 5 days of incubation, then the live and dead cells were stained with Calcein-AM/PI (keyGEN, China) double staining.

### 2.10 *In vivo* wound healing experiments

A full-thickness wound defect model was used to carry out *in vivo* wound healing experiments. Eight Sprague Dawley (SD) rats (female, 12-week-old, 220–250 g) were used to assess the hydrogel wound closure and healing performance. These research rats were first given a 7-day acclimatization period. For the surgical portion, aseptic procedures were used throughout. These rats were then given 30 mg/kg of pentobarbital sodium to make them unconscious. After that, the hair on their backs was partially removed, and a full-thickness wound (diameter: 5 mm) was made on it using a needle biopsy punch. Then, the wound on the left side of its back was left unattended as the blank control group (control group). And the wound on the right side of the back closed with the PEGDA/CS/CW hydrogel as the hydrogel experimental group (hydrogel group). Using a digital camera, the wound closure was evaluated on the third, sixth, and 12th days. At the same time, the new hydrogel dressings were changed. The reduction in wound area were calculated and normalized using the ImageJ software. And the wound healing rate (*R*_n_) was calculated according to Eq. 2.

$$R_{n}=\frac{S_{0}-S_{n}}{S_{0}}\times 100\%$$ (2)

*S*_n_ was the wound area on day *n* and *S*_0_ was the initial wound area.

We also evaluated the effect of hydrogel on wound healing by histological analysis. On day 3 and 12, cervical dislocation was used to euthanize experimental animals. The wound sites and encircling tissues were collected, fixed in a 10% paraformaldehyde solution and paraffin-embedded. Hematoxylin and eosin reagents (H&E, Beyotime, China) and Masson trichrome staining were used to color the tissue slices in order to observe the morphology, regeneration, and collagen deposition.

### 2.11 Statistical analysis

To ascertain whether or not the differences between the groups were statistically significant, statistical analysis was carried out. Data from independent parallel experiments were calculated using the mean standard deviation. The statistical analysis was performed using SPSS software. At *p < 0.05, significance was accepted; at **p < 0.01, statistical significance was higher; and at ***p < 0.001, significance was highest.

## 3 Results and discussion

### 3.1 Analysis of PEGDA characterization results

**FIGURE 2:**
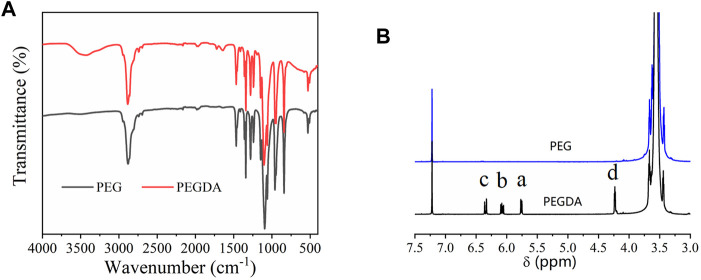
**(A)** FTIR spectra of PEG and PEGDA. **(B)** ^1^H NMR spectra of PEG and PEGDA.

The infrared spectra and 1H NMR spectra of PEG and PEGDA are shown in Figure 2. Figure 2A showed that PEG and PEGDA both had strong absorption peaks near 2,885 cm^−1^ generated by the stretching vibration of C-H bonds. Compared with PEG, PEGDA had two new characteristic absorption peaks at 1720 cm^−1^ and 1,190 cm^−1^, corresponding to the absorption peaks of the C=O bond and C-O-C bond respectively. These two vibration absorption peaks were characteristic peaks produced by the end group of polyethylene glycol after esterification by acyl chloride, which preliminarily proved that the end hydroxyl group of PEG was replaced by the acrylate group. The successful synthesis of PEGDA was further confirmed by 1H MNR spectrogram analysis (Figure 2B). Compared with PEG, PEGDA samples shown three distinct proton peaks (a, b, and c) in the range of 5.8–6.3 ppm, which were attributed to the three H proton peaks of the double bond on the PEGDA esterified by acrylic acid. In addition, there was an H proton peak (d) near 4.3 ppm, which corresponded to the hydrogen signal on the PEDGA carbon skeleton (-CH_2_O-CO-). The successful synthesis of polyethylene glycol diacrylate was confirmed.

### 3.2 Characterization of CW

**FIGURE 3:**
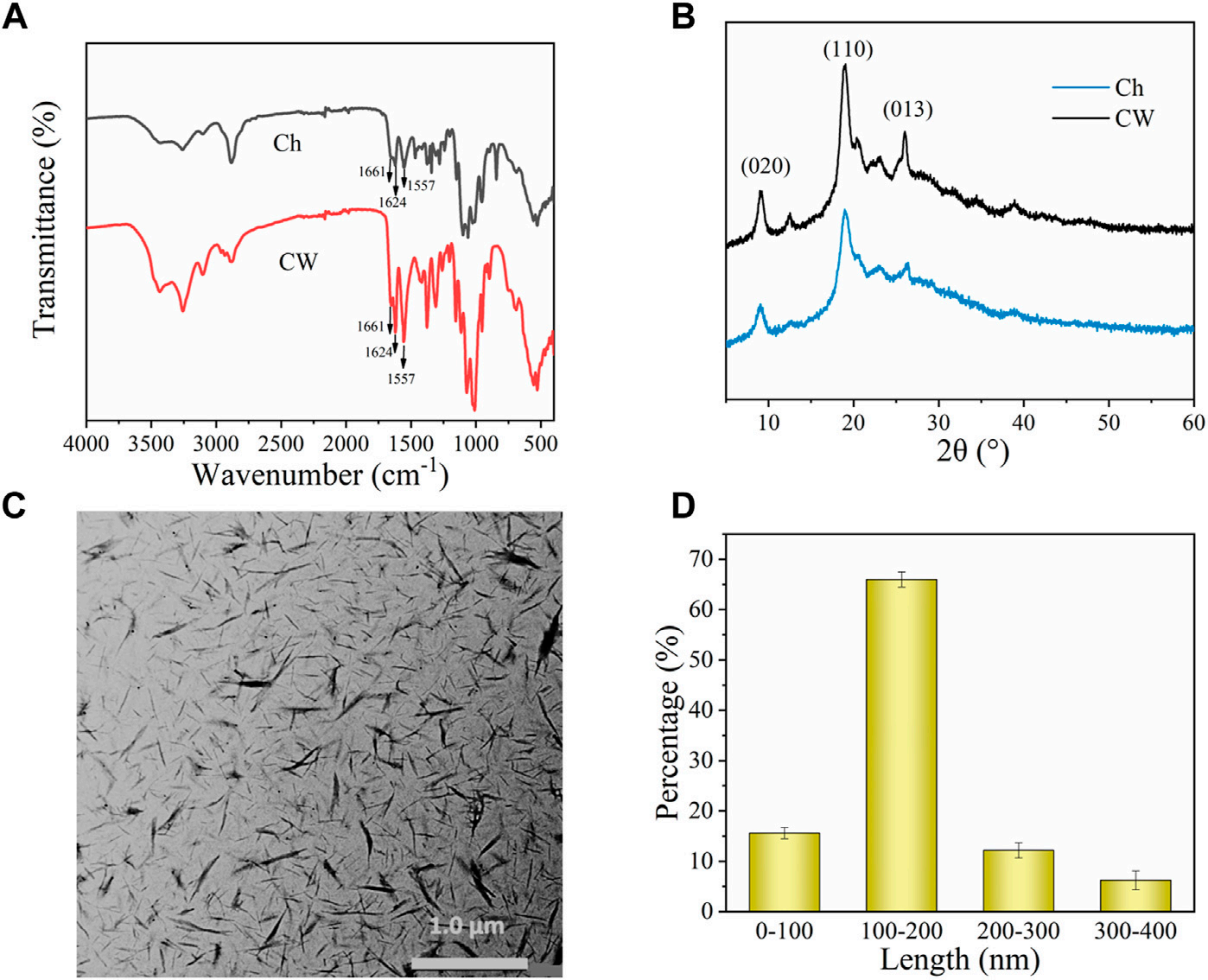
**(A)** FTIR spectra of Ch and CW. **(B)** XRD spectrum of Ch and CW. **(C)** Transmission electron microscopy pattern of CW. **(D)** The size distribution of CW.

The infrared spectra of Ch and CW (Figure 3A) showed that there were characteristic peaks in the amide I and amide II regions (1,661, 1,624, and 1,557 cm^−1^), which were characteristic of *α*-chitin. Compared with Ch, CW showed sharp characteristic peaks, which indicated that hydrochloric acid may change the structure of Ch and form highly crystalline whiskers (Phongying, Aiba, and Chirachanchai, 2007; Chang et al., 2010). It could be seen from the XRD patterns of Ch and CW (Figure 3B) with acid-hydrolysis that the main peaks at 9° and 19° (2θ) become sharper, which also confirmed the crystal structure of CW (Phongying, Aiba, and Chirachanchai, 2007).

TEM characterization confirmed the whisker morphology of CW. It could be observed from Figure 3C that the prepared CW was needle-like, with uniform size, spatial distribution, and no serious agglomeration phenomenon. In addition, we used the ImageJ software tool to measure 100 random chitin nano-whiskers in the SEM image and get the distribution of CW length. And the width distribution was between 10–40 nm. As could be seen from the CW size distribution diagram (Figure 3D), the CW length was concentrated in the interval of 100–200 nm (67%).

### 3.3 Adhesion behavior

**FIGURE 4:**
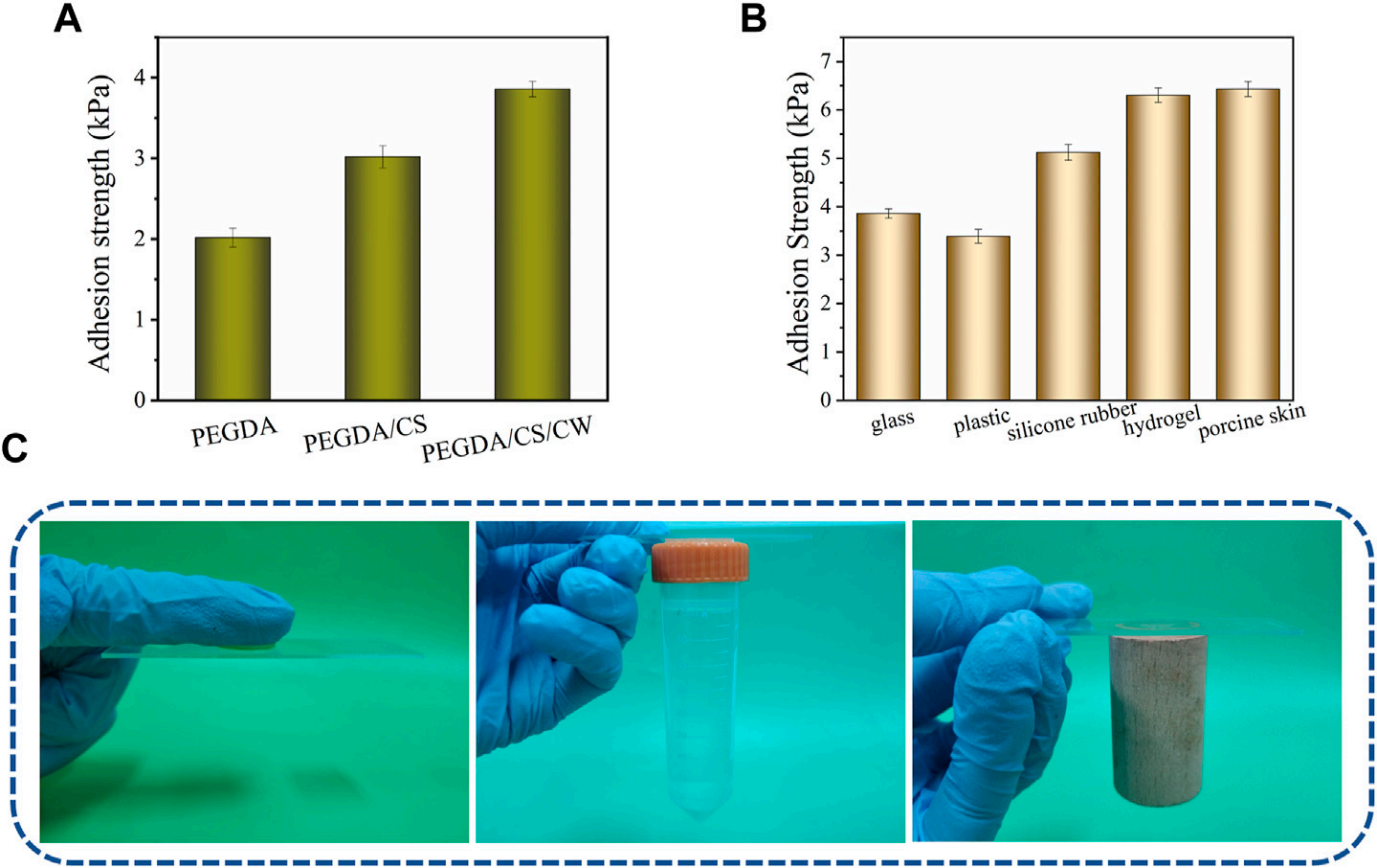
**(A)** Adhesion strength between hydrogel of different components and glass sheets. **(B)** PEGDA/CS/CW hydrogel adhesion strength to various materials. **(C)** PEGDA/CS/CW hydrogel adhesion to nitrile glove, plastic and wood.

As an emerging biomedical material of tissue engineering, adhesive hydrogel has attracted much attention in replacing or assisting traditional therapeutic methods, especially in the research of adhesion properties (Yang et al., 2022). In this paper, the adhesive property of hydrogel was evaluated by a lap shear test. Firstly, the adhesion strength between PEGDA, PEGDA/CS or PEGDA/CS/CW hydrogel and glass was investigated. As shown in Figure 4A, the adhesion strength between the above three hydrogel and glass sheets increased in turn, especially the PEGDA/CS/CW hydrogel displayed the highest adhesion strength of 4.2 kPa to glass sheets. PEGDA hydrogel with smooth surface and dense holes was adsorbed on the glass sheet like a suction cup, and the adhesion strength could reach 2.1 kPa. However, for rough and uneven materials, its adhesion property would completely fade away. CS is a non-toxic natural antibacterial polymer commonly used as a wound dressing (Yan et al., 2021). Addition of CS made hydrogel further increase interface adhesion through free diffusion through tissue interfaces, topological entanglement, and ion chelation, as if two interfaces were stitched together at the molecular scale (Li et al., 2017; Yang et al., 2018; Liang et al., 2021). With the introduction of CW, the mechanical strength and adhesion strength of hydrogel were further improved.

PEGDA/CS/CW hydrogel was tested for their adhesion to plastic, silicone rubber, hydrogel and pigskin. According to the results (Figure 4B), the adhesion strength of hydrogel for smooth and dense materials (glass and plastic) was relatively weak. But the adhesion performance of hydrogel was stronger for rough or conducive materials (hydrogel and porcine skin) which was benefited for CS diffusion. The PEGDA/CS/CW hydrogel could adhere to porcine skin up to 6.5 kPa, which was much better than medical fibrin adhesives (usually less than 4 kPa) (Deng et al., 2019; Guo et al., 2021). Figure 4C showed the adhesion between PEGDA/CS/CW hydrogel and three kinds of materials of nitrile gloves, plastics and wood.

### 3.4 Mechanical performance

**FIGURE 5:**
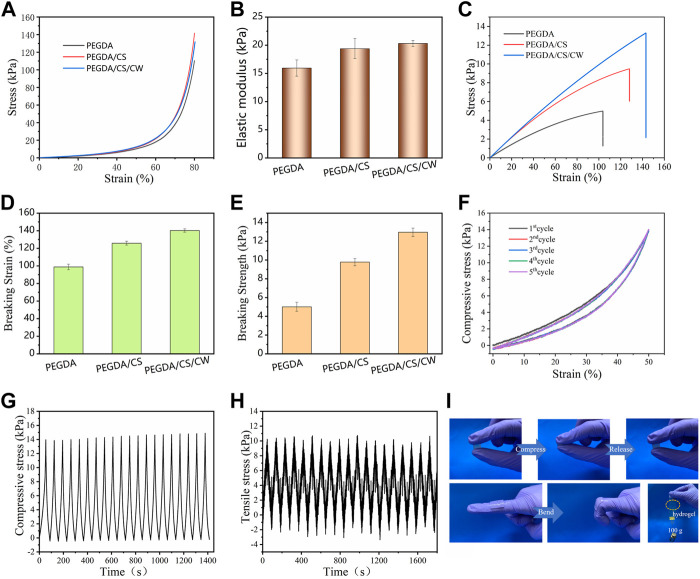
Mechanical properties of the hydrogel. **(A)** Compressive stress-strain curves. **(B)** Compressive elastic modulus. **(C)** Tensile stress-strain curves. **(D)** Breaking strain, and **(E)** breaking strength of PEGDA, PEGDA/CS and PEGDA/CS/CW hydrogel. **(F)** Cyclic compressive stress-strain curves. **(G)** Compressive cyclic stress-time and **(H)** cyclic tensile stress-time curves of the PEGDA/CS/CW hydrogel. **(I)** PEGDA/CS/CW hydrogel mechanical properties and adhesion performance display pictures.

The ideal wound dressing requires not only adequate adhesion strength but also appropriate mechanical properties. As can be seen from the compression test results (Figure 5A), the three hydrogels had good compression performance. It did not break when compressed to 80% of their height. With the introduction of CS and CW, the compressive elastic modulus of hydrogel gradually increased (Figure 5B). CS and CW also contributed to the improvement of hydrogel tensile properties. Compared with PEGDA hydrogel, the elongation at break of PEGDA/CS/CW hydrogel increased from 100% to 145%, and the breaking strength increased from 5.0 kPa to 11.2 kPa, showing excellent toughness (Figures 5C–E). At the same time, the improvement of the mechanical strength of hydrogel further promoted the adhesion of hydrogel to the material surface. Traditional dressings tend to have poor viscoelasticity, limiting the movement of the wound site when applied to the wound. The PEGDA/CS/CW hydrogel designed in this study showed remarkable toughness and resilience, that could withstand several cycles of compression and stretch (Figures 5F–H). Stress loss of hydrogel could be ignored, during 20 cyclic compression and tensile tests. As shown in Figure 5I, the PEGDA/CS/CW hydrogel was extremely compressed, it can be seen that the hydrogel quickly returned to its initial state when the compression load is removed. In addition, the hydrogel attached to the knuckle was free to move with the knuckle bend. The force of the hydrogel adhering to the glass sheet can support a 100 g weight. PEGDA/CS/CW hydrogel had excellent mechanical properties and adhesion properties, providing a strong guarantee for effective wound closure.

### 3.5 Swelling properties

**FIGURE 6:**
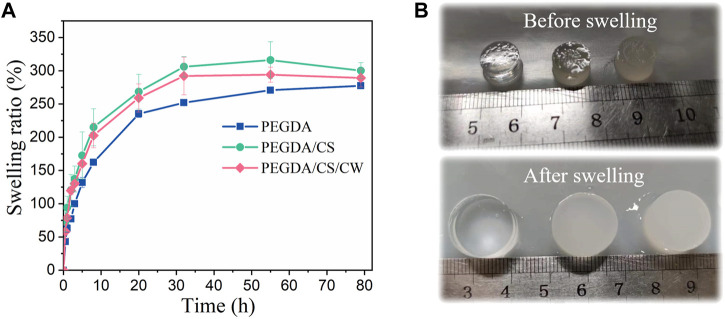
**(A)** Swelling rate of hydrogel. **(B)** Before and after pictures of hydrogel swelling.

According to the swelling test, PEGDA, PEGDA/CS, and PEGDA/CS/CW hydrogel all had high swelling rates, reaching 277%, 300%, and 289% respectively (Figure 6A). Proper suction capacity helps protect the tissue around the wound from exudate impregnation and reduces the risk of bacterial infection, which is a prerequisite for wound healing (Li et al., 2022). Hydrogel have a long time to reach swelling balance (50 h), which is conducive to the hydrogel to absorb the wound seepage, keep clean and moist of the wound for a long time, and promote wound healing. Figure 6B showed before and after the hydrogel swelling. The volume of hydrogel became larger after water absorption and swelling, but it did not burst, indicating that hydrogel showed good elasticity.

### 3.6 Cytotoxicity assay

**FIGURE 7:**
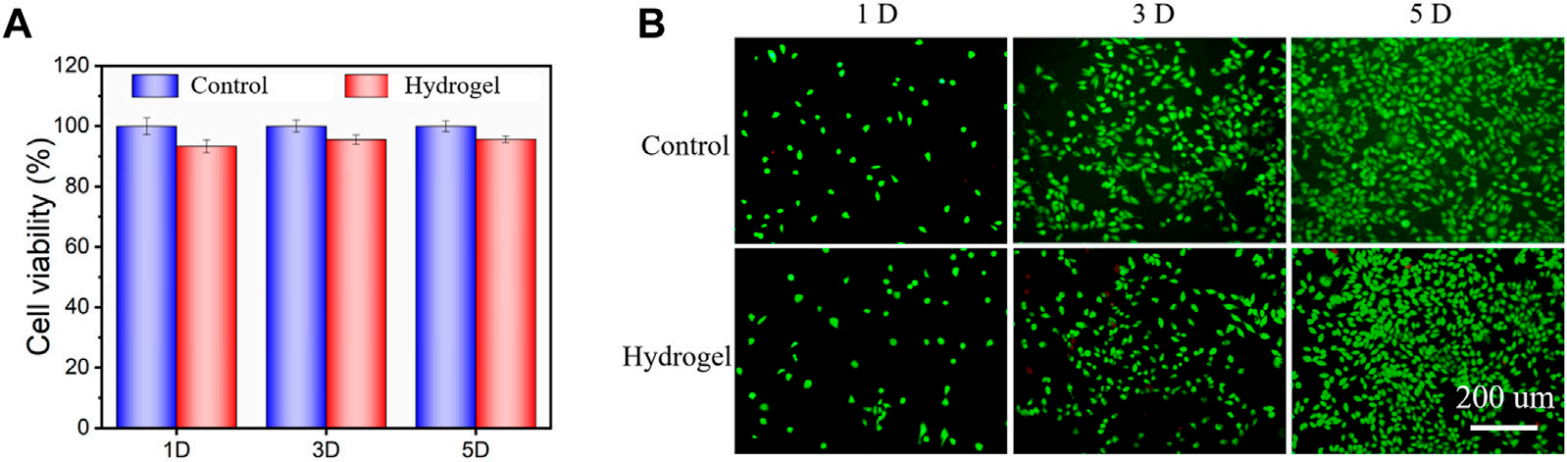
**(A)** Cell viability for the hydrogel was performed after 1, 3, and 5 days of culture. **(B)** Images of live/dead staining after culturing cells on the hydrogel.

Good biocompatibility is an important prerequisite for hydrogel to promote wound healing (Chen et al., 2018; Qu et al., 2018; Yang et al., 2021). In this experiment, the proliferation of 1, 3, and 5 days of cell culture was measured by CCK-8 kit. As shown in Figure 7A, the cell survival rate of the hydrogel group remained above 90%. In addition, it can be seen that the result of cell lived/dead staining (Figure 7B), the number of cells gradually increased with the increase of culture days. The cell morphology was saturated and spindle-shaped. In addition, the number of dead cells was small. The results of CCK-8 assay were consistent with the results of cell lived/dead staining, that indicated that the designed material had good cytocompatibility. Moreover, the PEGDA monomer had been completely reacted in the process of material synthesis. The introduction of SA and CW greatly improved the biocompatibility of materials.

### 3.7 Wound closure and healing

**FIGURE 8:**
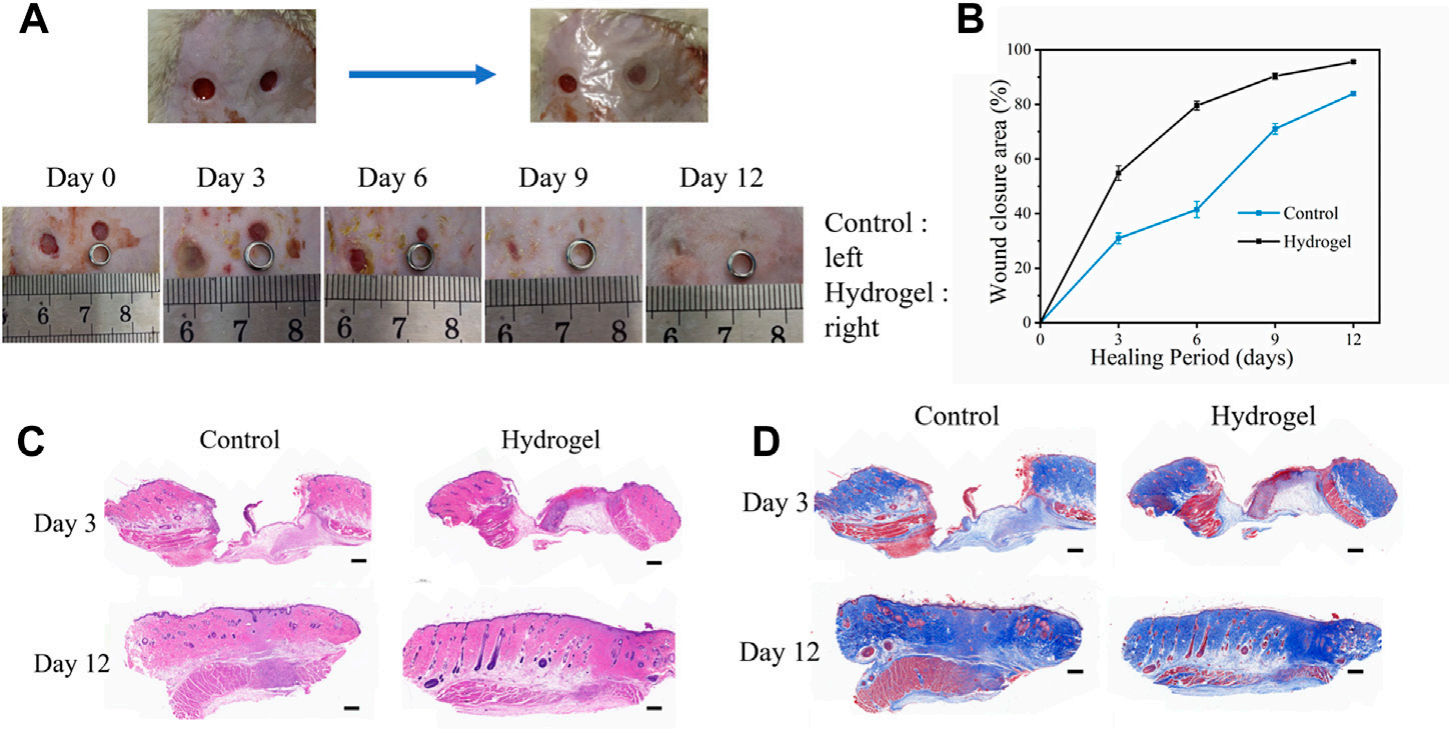
**(A)** Rat wound model and a general view of the appearance of the wound at different time points. **(B)** Wound healing ratio on day 3, 6, 9, and 12. **(C)** H&E and **(D)** Masson staining for different groups (scale: 500 μm).

To assess the therapeutic impact of hydrogel dressings on the cutaneous wound, a full-thickness wound defect model was created (Figure 8A). The effectiveness of wound treatment was assessed at the day 3, 6, 12 time periods, which was encouraged by *in vitro* results. The wound area clearly diminished throughout the course of the longer treatment periods in all groups. Particularly, the group treated with PEGDA/CS/CW dressing had noticeably quicker wound closure than the other group (Figure 8B). After 12 days of treatment, cutaneous wounds in the two group were almost healed and covered with neuroepithelium. The increased healing in the hydrogel dressing treated group was validated by the average wound closure ratio (Figure 8B), which was followed by PEGDA/CS/CW (95.6%) and control (83.9%). Our developed hydrogel dressing had a considerably faster rate of wound healing and a more beneficial therapeutic impact.

By H&E staining tissue section to observe the healing process of wounds, such as Figure 8C, wound healing on the third day, the two groups in skin wounds were larger, local thickening, significantly higher than the surface of the skin, the surface of the wound was large scab skin coverage, scab skin visible fill wound granulation tissue hyperplasia, granulation tissue within a large number of capillaries and inflammatory cells infiltration. But the wound of the hydrogel group healed better than the control group. The wound surface in the control group was still slightly higher than the skin on the 12th day of wound healing. The regeneration of the epidermis was gradually complete and the regeneration of glandular lumen in the dermis could be observed obviously. There were still slight inflammatory cell infiltration and disordered tissue arrangement in the wound. In the hydrogel group, the skin wounds healed completely, the regenerated glandular lumens and surrounding tissues were arranged neatly, and there was no obvious inflammatory cell infiltration around the wounds. As a result, when compared to the control group, the hydrogel group demonstrated superior wound repair.

Masson staining (Figure 8D) was used to evaluate collagen production at the wound site. On day 3 of wound healing, the control group and hydrogel group showed slight interstitial edema and accumulated a small amount of collagen, but the hydrogel group showed less interstitial edema and uniform distribution of collagen. On the 12th day of wound healing, collagen accumulation increased in each group, with the collagen accumulation in the control group being relatively loose, while the collagen accumulation in the hydrogel group was tightly arranged and orderly, with a large number of glandular lumens. The results obtained by the histological analysis were consistent with the wound healing process, indicating that PEGDA/CS/CW hydrogel had good wound closure and promoted repair.

In summary, the wound closure effect of the hydrogel group was better than that of control group. And at the same time, the hydrogel also provided a suitable mechanical environment for wound healing, aiding tissue repair and avoiding scarring. Most adhesives, including cyanoacrylates, dopamine hydrochloride adhesives, and aldehyde-based adhesives, effectively seal and heal most common wounds. However, those adhesives tend to deform or stretch away from the adhesive surface because they are not flexible enough (Liu et al., 2023). In addition, the hydrogel could also absorb wound exudate, keep the wound moist and clean for a long time, prevent bacterial infection, and facilitated the observation of wound healing.

## 4 Conclusion

Through the effective construction of DN hydrogel dressings in this work, we were able to demonstrate their significant potential for full-thickness skin wound healing. Compared with the aldehyde group or catecholic adhesive hydrogels reported in most literatures, they usually show weak interfacial toughness and high pH dependence (Liu et al., 2023). The tissue adhesion stress of the PEGDA/CS/CW hydrogel reached 6.5 kPa and the elongation was higher than 145%. The hydrogel can speed up wound healing while providing an ideal mechanical microenvironment for tissue regeneration and wound healing. Additionally, the transparency allows for easier clinical application and observation of wound healing. This hydrogel crosslinked by UV light is a promising material for wound treatment since it may be gelled in place enabling simple filling of uneven wounds.

## Data Availability

The original contributions presented in the study are included in the article/supplementary material, further inquiries can be directed to the corresponding authors.

## References

[B1] Annabi N. Kan Y. Ali T. Ali K. (2015). Elastic sealants for surgical applications. Eur. J. Pharm. Biopharm. 95, 27–39. 10.1016/j.ejpb.2015.05.022 26079524 PMC4591192

[B2] Chang A. Ye Z. Ye Z. Deng J. Lin J. Wu C. (2022). Citric acid crosslinked sphingan WL gum hydrogel films supported ciprofloxacin for potential wound dressing application. Carbohydr. Polym. 291, 119520. 10.1016/j.carbpol.2022.119520 35698364

[B3] Chang P. R. Jian R. Yu J. Ma X. (2010). Starch-based composites reinforced with novel chitin nanoparticles. Carbohydr. Polym. 80, 420–425. 10.1016/j.carbpol.2009.11.041

[B4] Chen G. Yu Y. Wu X. Wang G. Ren J. Zhao Y. (2018). Bioinspired multifunctional hybrid hydrogel promotes wound healing. Adv. Funct. Mater. 28, 1801386. 10.1002/adfm.201801386

[B5] Costa E. M. Silva S. Veiga M. Tavaria F. K. Pintado M. M. (2018). Chitosan’s biological activity upon skin-related microorganisms and its potential textile applications. World J. Microbiol. Biotechnol. 34, 93–96. 10.1007/s11274-018-2471-2 29900482

[B6] Dąbrowska A. K. Spano F. Derler S. Adlhart C. Spencer N. D. Rossi R. M. (2018). The relationship between skin function, barrier properties, and body-dependent factors. Skin Res. Technol. 24, 165–174. 10.1111/srt.12424 29057509

[B7] Deng J. Tang Y. Zhang Q. Wang C. Liao M. Ji P. (2019). A bioinspired medical adhesive derived from skin secretion of *Andrias davidianus* for wound healing. Adv. Funct. Mater. 29, 1809110. 10.1002/adfm.201809110

[B8] Dhivya S. Vijaya Padma V. Santhini E. (2015). Wound dressings–a review. BioMedicine 5, 22–25. 10.7603/s40681-015-0022-9 26615539 PMC4662938

[B9] Du X. Hou Y. Wu L. Li S. Ao Y. Kong D. (2020). An anti-infective hydrogel adhesive with non-swelling and robust mechanical properties for sutureless wound closure. J. Mater. Chem. B 8, 5682–5693. 10.1039/d0tb00640h 32500887

[B10] Du X. Wu L. Yan H. Qu L. Wang L. Wang X. (2019). Multifunctional hydrogel patch with toughness, tissue adhesiveness, and antibacterial activity for sutureless wound closure. ACS Biomaterials Sci. Eng. 5, 2610–2620. 10.1021/acsbiomaterials.9b00130 33405766

[B11] García F. Smulders M. J. (2016). Dynamic covalent polymers. J. Polym. Sci. Part A Polym. Chem. 54, 3551–3577. 10.1002/pola.28260 PMC5129565 27917019

[B12] Gonzalez A. C. de Oliveira T. F. C. de Araújo A. Z. Alena Ribeiro A. P. M. (2016). Wound healing-A literature review. An. Bras. Dermatol. 91, 614–620. 10.1590/abd1806-4841.20164741 27828635 PMC5087220

[B13] Guo Y. Wang Y. Zhao X. Xue L. Wang Q. Wen Z. (2021). Snake extract–laden hemostatic bioadhesive gel cross-linked by visible light. Sci. Adv. 7, eabf9635. 10.1126/sciadv.abf9635 34261653 PMC8279511

[B14] Hu D. Wu D. Huang L. Jiao Y. Li L. Lu L. (2018). 3D bioprinting of cell-laden scaffolds for intervertebral disc regeneration. Mater. Lett. 223, 219–222. 10.1016/j.matlet.2018.03.204

[B15] Hu X. Nian G. Liang X. Wu L. Yin T. Lu H. (2019). Adhesive tough magnetic hydrogels with high Fe3O4 content. ACS Appl. Mater. interfaces 11, 10292–10300. 10.1021/acsami.8b20937 30773877

[B16] Huang L. Zhu Z. Wu D. Gan W. Zhu S. Li W. (2019). Antibacterial poly (ethylene glycol) diacrylate/chitosan hydrogels enhance mechanical adhesiveness and promote skin regeneration. Carbohydr. Polym. 225, 115110. 10.1016/j.carbpol.2019.115110 31521272

[B17] Kim I. Y. Seo S. J. Moon H. S. Yoo M. K. Park I. Y. Kim B. C. (2008). Chitosan and its derivatives for tissue engineering applications. Biotechnol. Adv. 26, 1–21. 10.1016/j.biotechadv.2007.07.009 17884325

[B18] Kong D. Zhang Q. You J. Cheng Y. Cheng H. Chen Z. (2020). Adhesion loss mechanism based on carboxymethyl cellulose-filled hydrocolloid dressings in physiological wounds environment. Carbohydr. Polym. 235, 115953. 10.1016/j.carbpol.2020.115953 32122489

[B19] Ladet S. David L. Domard A. (2008). Multi-membrane hydrogels. Nature 452, 76–79. 10.1038/nature06619 18322531

[B20] Li J. Celiz A. D. Yang J. Yang Q. Wamala I. Whyte W. (2017). Tough adhesives for diverse wet surfaces. Science 357, 378–381. 10.1126/science.aah6362 28751604 PMC5905340

[B21] Li Y. Zhang Y. Wang Y. Kun Y. Hu E. Lu F. (2022). Regulating wound moisture for accelerated healing: A strategy for the continuous drainage of wound exudates by mimicking plant transpiration. Chem. Eng. J. 429, 131964. 10.1016/j.cej.2021.131964

[B22] Li Z. Milionis A. Zheng Y. Yee M. Codispoti L. Tan F. (2019). Superhydrophobic hemostatic nanofiber composites for fast clotting and minimal adhesion. Nat. Commun. 10, 5562–5611. 10.1038/s41467-019-13512-8 31804481 PMC6895059

[B23] Liang Y. He J. Guo B. (2021). Functional hydrogels as wound dressing to enhance wound healing. ACS Nano 15, 12687–12722. 10.1021/acsnano.1c04206 34374515

[B24] Liu H. Hu X. Wen L. Zhu M. Tian J. Li L. (2023). A highly-stretchable and adhesive hydrogel for noninvasive joint wound closure driven by hydrogen bonds. Chem. Eng. J. 452, 139368. 10.1016/j.cej.2022.139368

[B25] Minh N. C. Schwarz S. Stevens W. F. Si Trung T. (2019). Preparation of water soluble hydrochloric chitosan from low molecular weight chitosan in the solid state. Int. J. Biol. Macromol. 121, 718–726. 10.1016/j.ijbiomac.2018.10.130 30339999

[B26] Montagna W. (2012). The structure and function of skin. Netherlands: Elsevier.

[B27] Okuyama K. Noguchi K. Miyazawa T. Yui T. Ogawa K. (1997). Molecular and crystal structure of hydrated chitosan. Macromolecules 30, 5849–5855. 10.1021/ma970509n

[B28] Patil P. Russo K. A. McCune J. T. Pollins A. C. Cottam M. A. Dollinger B. R. (2022). Reactive oxygen species–degradable polythioketal urethane foam dressings to promote porcine skin wound repair. Sci. Transl. Med. 14, eabm6586. 10.1126/scitranslmed.abm6586 35442705 PMC10165619

[B29] Phillips T. J. Palko M. J. Bhawan J. (1994). Histologic evaluation of chronic human wounds treated with hydrocolloid and nonhydrocolloid dressings. J. Am. Acad. Dermatology 30, 61–64. 10.1016/s0190-9622(94)70009-5 8277033

[B30] Phongying S. Aiba S. Chirachanchai S. (2007). Direct chitosan nanoscaffold formation via chitin whiskers. Polymer 48, 393–400. 10.1016/j.polymer.2006.10.049

[B31] Pitpisutkul V. Prachayawarakorn J. (2022). Hydroxypropyl methylcellulose/carboxymethyl starch/zinc oxide porous nanocomposite films for wound dressing application. Carbohydr. Polym. 298, 120082. 10.1016/j.carbpol.2022.120082 36241320

[B32] Qu J. Zhao X. Liang Y. Zhang T. Ma P. X. Guo B. (2018). Antibacterial adhesive injectable hydrogels with rapid self-healing, extensibility and compressibility as wound dressing for joints skin wound healing. Biomaterials 183, 185–199. 10.1016/j.biomaterials.2018.08.044 30172244

[B33] Sahariah P. Másson M. (2017). Antimicrobial chitosan and chitosan derivatives: A review of the structure–activity relationship. Biomacromolecules 18, 3846–3868. 10.1021/acs.biomac.7b01058 28933147

[B34] Vakalopoulos K. A. Wu Z. Leonard K. Kleinrensink G. J. Johannes J. Vendamme R. (2015). Mechanical strength and rheological properties of tissue adhesives with regard to colorectal anastomosis: An *ex vivo* study. Ann. Surg. 261, 323–331. 10.1097/sla.0000000000000599 24670843

[B35] Vig K. Chaudhari A. Tripathi S. Dixit S. Sahu R. Pillai S. (2017). Advances in skin regeneration using tissue engineering. Int. J. Mol. Sci. 18, 789. 10.3390/ijms18040789 28387714 PMC5412373

[B36] Xu J. Liu Y. Hsu S. (2019). Hydrogels based on Schiff base linkages for biomedical applications. Molecules 24, 3005. 10.3390/molecules24163005 31430954 PMC6720009

[B37] Yan D. Li Y. Liu Y. Li N. Zhang X. Yan C. (2021). Antimicrobial properties of chitosan and chitosan derivatives in the treatment of enteric infections. Molecules 26, 7136. 10.3390/molecules26237136 34885715 PMC8659174

[B38] Yang J. Bai R. Suo Z. (2018). Topological adhesion of wet materials. Adv. Mater. 30, 1800671. 10.1002/adma.201800671 29726051

[B39] Yang J. Yu H. Wang L. Liu J. Liu X. Hong Y. (2022). Advances in adhesive hydrogels for tissue engineering. Eur. Polym. J. 172, 111241. 10.1016/j.eurpolymj.2022.111241

[B40] Yang Z. Huang R. Zheng B. Guo W. Li C. He W. (2021). Highly stretchable, adhesive, biocompatible, and antibacterial hydrogel dressings for wound healing. Adv. Sci. 8, 2003627. 10.1002/advs.202003627 PMC8061386 33898178

[B41] Zeng Z. Zhu M. Chen L. Zhang Y. Lu T. Deng Y. (2022). Design the molecule structures to achieve functional advantages of hydrogel wound dressings: Advances and strategies. Compos. Part B Eng. 247, 110313. 10.1016/j.compositesb.2022.110313

[B42] Zhang L. Liu M. Zhang Y. Pei R. (2020). Recent progress of highly adhesive hydrogels as wound dressings. Biomacromolecules 21, 3966–3983. 10.1021/acs.biomac.0c01069 32960043

